# Supplementary material for: Force-Driven Model for Automated Clear Aligner Staging Design Based on Stepwise Tooth Displacement and Rotation in 3D Space
Source: Bioengineering (Basel). 2025 Jan 25;12(2):111. doi: 10.3390/bioengineering12020111 (PMC11852307; doi:10.3390/bioengineering12020111)
Supplement: Supplementary file 1 [file bioengineering-12-00111-s001.zip › Ref31_Manual staging for a molar distalization case.html]

 

|

- 语言

- 深色主题
- 浅色主题
- 迪士尼主题-蜘蛛侠
- 迪士尼主题-艾莎

- 中文
- 英文
- 西班牙
- 日语
- 法语
- 繁体
- 德语
- 越南语
- 葡萄牙语

- 托槽转矩

0

0

0

0

0°

0°

--

查看更多 >>

- --0.0mm
- --0.0mm
- --0.0mm
- --0°
- --0°
- --0°

调整

SML

GIF

WebM

MP4

HTML

-

78.8% ± 1.72%

91.5% ± 1.51%

12

11

21

22

12

11

21

22

13-23

14-24

15-25

16-26

43-33

44-34

45-35

46-36

转矩托槽数据

上颌-牙位号

提交单托槽转矩

目标位托槽转矩

下颌-牙位号

提交单托槽转矩

目标位托槽转矩

病例备注仅供医生自己保存备忘，与设计相关沟通请使用动画反馈、预约功能。

1

2

3

4

5

6

7

8

0

A

S

D

V

Ctrl + Z

Ctrl +Shift + Z

Ctrl + R

X

正颌色块图

 
